# Flexural Strength of Different Restorative Materials Used for Direct Restoration in Pediatric Dentistry: An In Vitro Study

**DOI:** 10.3390/biomimetics11010016

**Published:** 2025-12-29

**Authors:** Ioana Elena Lile, Carolina Cojocariu, Ciprian Pasca, Andra-Alexandra Stăncioiu, Luminiţa Ligia Vaida, Diana Marian

**Affiliations:** 1 Department of Dentistry, Faculty of Dentistry, “Vasile Goldis,” Western University of Arad, 94-96 Revolutiei Blvd., 310025 Arad, Romania; lile.ioana@uvvg.ro (I.E.L.); cojocariu.carolina@uvvg.ro (C.C.); marian.diana@uvvg.ro (D.M.); 2 Department of Dentistry, Faculty of Medicine and Pharmacy, University of Oradea, 1 Universitateai Street, 410087 Oradea, Romania; ligia_vaida@uoradea.ro

**Keywords:** pediatric dentistry, composite resins, glass ionomer cements, resin-modified glass ionomers, flexural strength, minimally invasive dentistry

## Abstract

Background: Preservation of tooth structure is a key principle in pediatric dentistry, where restorative materials must balance mechanical strength with the preservation of pulp vitality and minimally invasive techniques. The aim of this in vitro study, as it relates to pediatric dentistry, was to investigate the flexural strength of common composite resins, glass ionomer cements, and resin-modified glass ionomer cement within standardized and homogeneous laboratory conditions. Methods: This study evaluated the flexural strength of seven restorative materials: four composites (Filtek™ Z250, Filtek™ Supreme XT, Gradia, Premise), two GICs (Ketac™ Molar Easymix, GC Fuji IX GP), and one RMGIC (Vitremer). Standardized specimens were prepared and tested using a three-point bending protocol with a universal testing machine (Zwick-Roell Z005). A total of 49 specimens were fabricated and analyzed. Statistical analysis was performed with a one-way ANOVA followed by Tukey’s post hoc test. Results: The flexural strength value of composite resins was significantly greater than that of the glass ionomer and resin-modified glass ionomer cements (*p* < 0.001). Filtek™ Z250 had the highest flexural strength, and Vitremer, a resin-modified glass ionomer cement, exhibited intermediate performance. Ketac™ Molar Easymix had the lowest values among conventional glass ionomer cements, whilst the flexural strength values obtained for GC Fuji IX GP were similar to some composite materials but with higher variability. Conclusions: Composite resins remain the most durable option for pediatric restorations in stress-bearing areas, whereas RMGICs provide a compromise between mechanical performance and biological advantages such as fluoride release and biocompatibility. Conventional GICs, despite their lower flexural strength, retain clinical relevance in low-load sites and for patients at a high risk of caries. Material selection in pediatric dentistry should therefore be tailored to the child’s age, tooth location, and functional demands to ensure long-lasting, minimally invasive restorations. This study involved only mechanical properties alone, and biological aspects, such as fluoride release and biocompatibility, were not considered. Material selection in pediatric dentistry should therefore take into account mechanical requirements, restorative location, and clinical environment.

## 1. Introduction

The principles of long-term teeth preservation with minimally invasive restorative therapy have become paramount in contemporary dentistry, with importance in pediatric dentistry since pulp vitality and the long-term activity of teeth is critically required [1,2]. Because of the biological and clinical specificity of primary and young permanent teeth, tooth restoration materials applied in children should consequently have good mechanical reliability. Periodic improvements in dental restorative materials have been substantial over the past 50 years. The development of bis-GMA based systems was followed by the development of composite resins, which improved the mechanical and esthetic properties with years [3,4,5].

Glass ionomer cements (GICs) were also created, with chemical attachment to the tooth structure and fluoride release provided, with biological and preventative benefits, especially in children [6]. Subsequently, resin-modified glass ionomer cements (RMGICs) were developed to realize resin-based materials for superior handling and mechanical properties in combination with the bioactive properties from standard GICs [7,8].

In current pediatric practice, composite resins, GICs, and RMGICs are employed universally for direct restorations, each having unique advantages and limitations. Composite resins are usually mechanically superior and are used in stress-bearing areas, but in pediatric patients their clinical performance might be hindered by the requirement for stringent moisture control. This is often hard to obtain due to decreased patient cooperation and a limited treatment time [9,10,11,12]. They can be chemically bonded and release fluoride, while showing lower strength of mechanical reinforcement in contrast to composites, hence reducing their applicability in load-bearing restorations [13,14,15,16]. RMGICs are an intermediate category, striving to provide some mechanical performance and biological benefits as simultaneously as possible [17,18]. Restorative materials used in pediatric dentistry are subjected to masticatory forces that produce complex stress profiles that include tensile, compressive, and shear components. Flexural strength has become well-established as a clinically relevant mechanical parameter, as it determines a material’s ability to withstand fracture under such combined loading conditions [19,20]. Flexural strength testing is commonly used to evaluate the mechanical integrity of restorative materials under the same laboratory conditions as international standards such as ANSI/ADA specifications [2,21,22]. Therefore, flexural strength provides a useful reference to predetermine its performance in clinical situations, in particular for restorations subjected to occlusal forces. Despite there being substantial research on each individual restorative material group, a clear dearth of the literature exists. Most studies either test composites, GICs, or RMGICs individually or use mixed testing procedures, making direct comparison difficult. There has been little comparison conducted under the same or equivalent experimental conditions, in particular for the most widely used materials in pediatric dentistry [23,24,25].

The ability of restorative materials to withstand masticatory forces makes flexural strength one of their most important mechanical properties for successful restorations [24]. The restorations in pediatric teeth need to function properly for multiple years until the permanent teeth develop their full structure [25]. The selection of materials for dental restorations depends on standardized laboratory tests that evaluate mechanical behavior under controlled conditions [26,27,28].

The absence of consistent comparison data could impede the evidence-based material choice and clinical decision making.

Thus, the objective of this in vitro study was to compare the flexural strength of generally practiced composite resins and glass ionomer cements with a resin-modified glass ionomer cement for the same and standard laboratory conditions through a three-point bending assay. It was postulated that composite resins will have superior flexural strength compared with conventional GICs, while RMGICs have demonstrated intermediate mechanical performance. By offering standardized comparative data, this study aims to aid the clinically informed choice of restorative materials for pediatric dental applications.

## 2. Materials and Methods

### 2.1. Materials

This in vitro experimental research examined seven pediatric dental restorative materials that belonged to three groups: composite resins (*n* = 4), conventional glass ionomer cements (*n* = 2), and a resin-modified glass ionomer cement (*n* = 1). The restorative materials commonly used in pediatric dentistry were selected: four composite resins, two conventional glass ionomer cements (GICs), and one resin-modified glass ionomer cement (RMGIC). All materials were handled strictly according to the manufacturers’ instructions regarding mixing, insertion, and curing. A total of 49 specimens were fabricated (*n* = 7 per material). The tested materials, their codes, manufacturers, and compositions are listed in Table 1.

The study included well-established restorative systems that remain in clinical use, allowing a comparative assessment under standardized laboratory conditions. The purpose was not to introduce novel formulations but to provide consistent benchmark data across commercially available materials currently used in pediatric restorative care.

Composites:

- Filtek™ Z250 (3M ESPE, St. Paul, MN, USA)—microhybrid composite
- Filtek™ Supreme XT (3M ESPE, USA)—nanocomposite
- GC Gradia (GC Corporation, Tokyo, Japan)—microfilled hybrid composite
- Premise (Kerr Corporation, Orange, CA, USA)—nanocomposite

Glass ionomer cements (GICs):

- Ketac™ Molar Easymix (3M ESPE, USA)
- GC Fuji IX GP (GC Corporation, Japan)

Resin-modified glass ionomer cement (RMGIC):

- Vitremer (3M ESPE, USA)

### 2.2. Prototype and Mold Fabrication

Forty-nine specimens were fabricated, seven for each group of materials. The sample size was small, but pilot data suggested power to see real differences in flexural strength among material groups. A post hoc power analysis (alpha = 0.05 effect size f = 0.85 from pilot data) showed a power of 0.82 for detecting differences among material groups.

**Figure 1:**
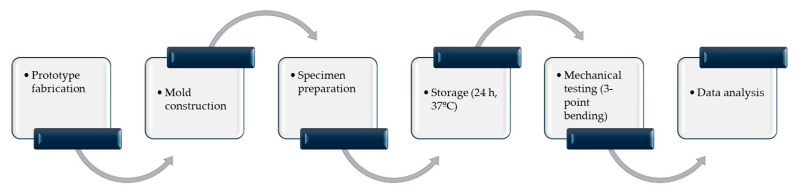
Experimental workflow of specimen fabrication, storage, flexural strength testing, and data analysis.

Specimens were designed as rectangular prisms. The following evaluation process followed ANSI/ADA Specification No. 27 guidelines by placing specimens under testing equipment with a 20 mm support span between the lower bars [29]. The standardized geometry (25 mm length, 2 mm width, 2 mm height) and mold design ensured dimensional consistency prior to specimen preparation and mechanical testing. Figure 1 summarizes the overall experimental workflow from specimen fabrication to mechanical testing and data analysis.

### 2.3. Specimen Preparation

**Figure 2:**
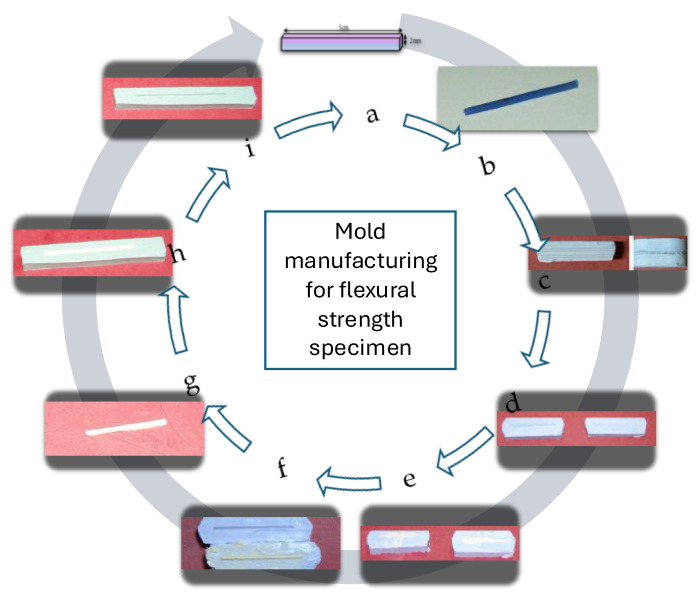
(**a**) Schematic of the rectangular prism prototype; (**b**) wax prototype for flexural strength testing; (**c**) wax prototype encased in plaster mold; (**d**) mold; (**e**) condensation of self-curing acrylic resin; (**f**) acrylic prototypes; (**g**) finishing and polishing of the acrylic prototype prior to impression taking; (**h**) impression of acrylic prototype; (**i**) silicone template mold for flexural strength specimen fabrication.

To produce standardized specimens, wax prototypes were first fabricated, then reproduced in self-curing acrylic resin. Final impressions served as the definitive templates for specimen fabrication (Figure 1 and Figure 2).

Composite resins: The materials were inserted incrementally (2 mm layers) into silicone molds. Each increment (~10 mm in length) was polymerized for 40 s using an LED curing unit (Bluephase, Ivoclar Vivadent, Liechtenstein) at 1200 mW/cm^2^, with overlapping irradiation zones to ensure complete polymerization along the specimen length.

Composite resins were manually inserted step by step, as is standard laboratory protocol in the field of restorative material testing. To minimize variability in layer thickness, silicone molds with standardized dimensions were used, and each increment was managed to achieve an approximate thickness of 2 mm. To promote uniform polymerization along the entire specimen length, overlapping light-curing zones and uniform curing times were put in place.

Glass ionomer cements (GICs): Powders and liquids were mixed by hand following the manufacturer’s instructions, placed in Vaseline-coated molds, and gently vibrated to reduce porosity. A glass slide was applied to achieve uniform density.

Resin-modified glass ionomer cement (RMGIC): This was prepared similarly to the GICs, followed by light-curing in 1 cm segments for 40 s to ensure full polymerization of the resin component.

Small internal voids might work as stress intensifiers and could lead to more variability in measuring flexural strength. But, since the same preparation method and acceptance rules were used for all material groups, any possible effect they have should be seen as general rather than specific to a certain group.

**Figure 3:**
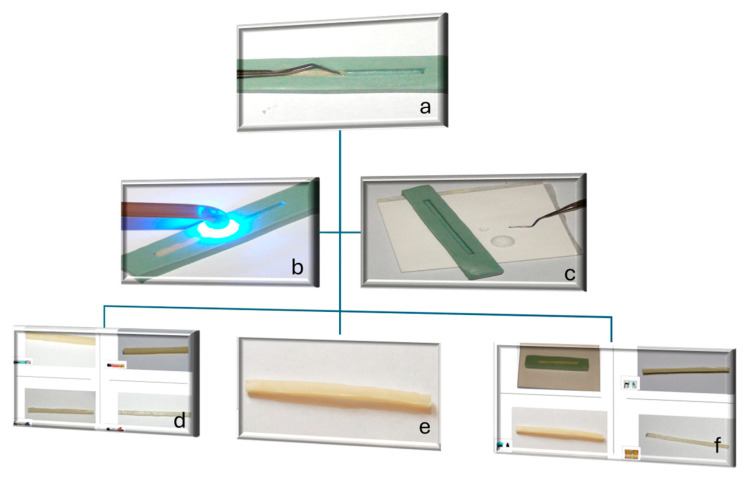
(**a**) Incremental insertion of composite resin into the mold; (**b**) light-curing of composite resin increment; (**c**) fabrication of glass ionomer cement specimen; (**d**) finished composite resin specimens; (**e**) finished specimens; (**f**) finished glass ionomer cement specimens.

After curing, all specimens were trimmed and measured with a digital caliper (±0.01 mm) to confirm dimensions within ±0.2 mm tolerance. Minor internal voids (<0.5 mm) were accepted as being inherent to manual preparation. After curing and finishing, the surfaces of all specimens were polished with 600- and 1200-grit silicon carbide papers under water to remove surface irregularities and ensure uniform contact during testing (Figure 3).

### 2.4. Storage Conditions

Specimens were stored in distilled water at 37 °C for 24 h before testing. Specimens were kept under distilled water in sealed containers in a laboratory incubator, achieving stable and physiologically relevant temperatures. The temperature was set by continuous monitoring and checked periodically by the incubator’s internal digital monitoring system. The storage process simulated intraoral hydration and allowed maturation, enabling the full development of acid–base reactions in the GIC and RMGIC groups and the composite resins to reach the water absorption equilibrium, and the storage period enabling the relaxation of stresses during polymerization or setting processes.

### 2.5. Flexural Strength Testing

Mechanical testing was carried out on a universal testing machine (Zwick-Roell Z005, Ulm, Germany) using three-point bending.

**Figure 4:**
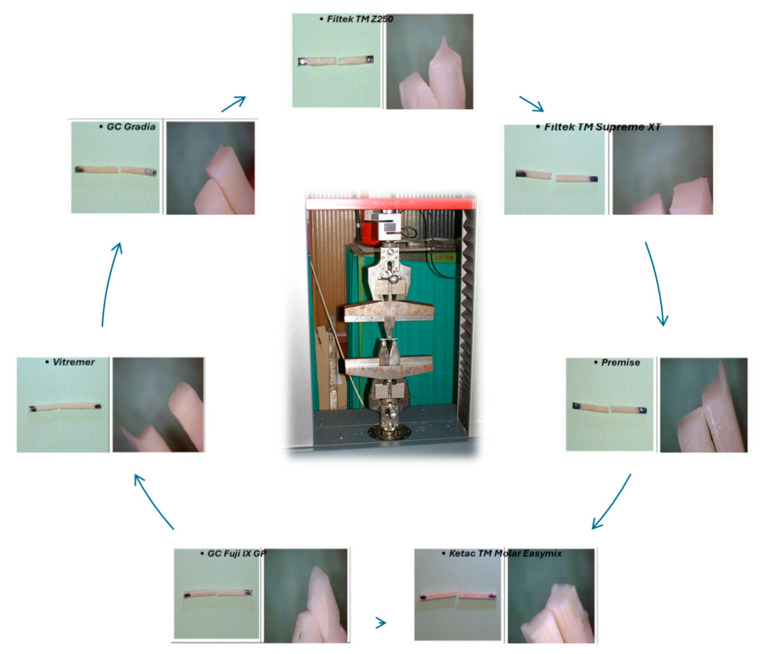
Representative images of each material class before and after mechanical testing.

- Setup: Specimens were placed on two rounded lower supports, so that the specimens rested under the testing equipment with 20 mm lower bar spacing and a specimen length of 25 mm, following ANSI/ADA Specification No. 27 guidelines. A rounded upper loading nose applied force at the midpoint.
- Loading: The crosshead speed was 0.75 mm/min until fracture occurred (Figure 4). The fracture line in all studied specimens followed the path of the applied force, which created a straight boundary that divided the specimen into two parts (Figure 4).
- Data recording: The maximum load (F, N) was automatically registered.

Flexural strength (*σ*, in MPa) was calculated using the standard formula:

(1) $$\sigma =\frac{3FL}{2bh^{2}}$$

where *F* = maximum load at fracture (N), *L* = support span (mm), *b* = specimen width (mm), and *h* = specimen height (mm).

### 2.6. Data Handling

Each of the 49 specimens was tested individually. Results were expressed as mean ± standard deviation for each group. Due to the limited sample size (*n* = 7 per material), statistical power was modest, and findings should be interpreted with caution regarding generalizability.

### 2.7. Statistical Analysis

Statistical analyses were performed using SPSS version 26.0 (IBM Corp., Armonk, NY, USA). Descriptive statistics (mean ± standard deviation) were computed for each group. Data normality was verified using the Shapiro–Wilk test. Differences among the seven material groups were evaluated using a one-way ANOVA, followed by Tukey’s post hoc test (*p* < 0.05).

Sample size calculation. A sensitivity analysis was conducted using G*Power 3.1 (Heinrich Heine University, Düsseldorf, Germany) for a one-way ANOVA (α = 0.05, total *N* = 49). The analysis showed that the study achieved 80% power to detect large effect sizes (Cohen’s f ≈ 0.45–0.50), indicating that subtle differences might not be detected with the current sample size. Consequently, the present findings should be regarded as exploratory and hypothesis-generating.

## 3. Results

The testing process resulted in the successful fracture of all 49 fabricated specimens and did not require any specimen exclusion because of equipment failure or testing mistakes. The specimens failed through their middle section during testing, which confirmed proper three-point bending failure without any early edge breakdown. The mean flexural strength values and standard deviations for all tested materials are presented in Table 2. Composite resins demonstrated the highest flexural strength, with Filtek Z250 (32.1 ± 2.4 MPa) performing best, followed by Filtek Supreme XT (17.3 ± 1.8 MPa), Premise (16.8 ± 2.0 MPa), and Gradia (8.2 ± 1.1 MPa). The resin-modified glass ionomer cement (Vitremer) achieved an intermediate value (12.5 ± 1.7 MPa). Among the glass ionomer cements, Ketac™ Molar Easymix (4.3 ± 0.6 MPa) showed the lowest resistance, whereas GC Fuji IX GP (18.4 ± 3.2 MPa) demonstrated a moderate flexural strength, outperforming Gradia (8.2 ± 1.1 MPa) and approaching the lower range of composite materials.

The Levene’s test results showed that the variance between groups was equal, the F-value was 1.95, and the *p*-value reached 0.082.

One-way ANOVA: The seven material groups showed significant differences in flexural strength according to the one-way ANOVA analysis (F_6,42_ = 87.34, *p* < 0.001, η^2^ = 0.926). The large effect size (η^2^ = 0.926) shows that material type explains 92.6% of the total variance in flexural strength.

Post hoc comparisons: The Tukey’s HSD test results showed the following significant differences between groups.

These seven restorative materials exhibited statistically significant differences in their flexural strength (one-way ANOVA, *p* < 0.001). The composite resins showed the greater flexural strengths. Filtek™ Z250 exhibited the highest fracture resistance in terms of flexural strength, being approximately seven times larger than Ketac™ Molar Easymix, and more than twice as large as Gradia. Resin-modified glass ionomer cement (Vitremer) exhibited intermediate mechanical performance, with levels of flexural strength being about 40–45% lower compared to high-strength composite resins but considerably larger than Ketac™ Molar Easymix (*p* < 0.001). The flexural strength of GC Fuji IX GP was found to be similar to some of the composite materials, when compared to Filtek™ Supreme XT and Premise (*p* > 0.05), with Ketac™ Molar Easymix showing the lowest flexural strength. Post hoc Tukey’s analysis confirmed major differences between composites and conventional glass ionomer cements (*p* < 0.001), but also pointed to material-based variability between the various material systems. A total of at least a twofold increase in flexural strength was found for composite resins when compared with conventional glass ionomer cements.

## 4. Discussion

The present study compared the flexural strength of restorative materials that are widely used in pediatric dentistry, including composite resins, a resin-modified glass ionomer cement (RMGIC), and conventional glass ionomer cements (GICs), under standardized laboratory conditions. The results revealed a distinct hierarchy: composite resins exhibited the highest flexural strength, RMGIC showed intermediate values, and conventional GICs demonstrated the lowest performance. These findings are consistent with the results of previous investigations [2,18,19,20,21,22,23,24,25,26,27] and reinforce the clinical trend toward minimally invasive and biologically oriented restorative approaches in pediatric dentistry.

This study demonstrated that pediatric restorative materials show different flexural strength levels, where composite resins achieve better results than glass ionomer cements and a resin-modified glass ionomer cement. The experimental findings match the natural differences that exist between the materials because of their chemical makeup and their internal structure. The composite resins achieve their improved flexural strength because their resin matrix contains 60–80% inorganic filler particles, which create a dense structure. The resin matrix that contains Bis-GMA, and UDMA and TEGDMA monomers distributes stress to the filler particles that carry most of the weight. The mechanical strength of the Filtek™ Z250 material increases because its high filler content creates a stronger composite structure, which reduces the polymer phase weakness and produces a more rigid material. The process of silanization on filler particles creates better connections between fillers and matrix materials, which enables successful stress distribution and reduces the occurrence of interface breakdowns. The flexural strength of conventional glass ionomer cements remained lower than for other materials because their setting process and tiny structural elements created these differences. The acid–base reaction in GICs creates a salt-bridge matrix that provides chemical bonding and fluoride release but results in a less-dense structure that is weaker than that of polymer-based composites. The flexural strength of the Ketac™ Molar Easymix remains low because of the mechanical limitations that traditional GIC formulations present. The GC Fuji IX GP shows a better performance than the Ketac™ Molar Easymix because it approaches the strength of composite materials, which demonstrates that modern GIC formulation improvements lead to better mechanical properties. Vitremer (RMGIC) shows average results because it combines traditional GIC acid–base chemistry with light-activated or chemical resin polymerization methods. The dual-setting method produces better mechanical properties than traditional GICs while preserving their essential properties, which include fluoride release and chemical bonding ability. The mechanical improvement in RMGICs results from a reinforcing polymer network that exists inside the glass ionomer matrix to reduce porosity and create a stronger bond between components. The GC Fuji IX GP shows greater variability than composite resins according to the observed results. The setting process of glass ionomer cements becomes more sensitive to manipulation factors, which include the powder-to-liquid ratio, mixing techniques, and environmental settings. The mechanical properties of the material become significantly affected by small changes in these parameters, which result in the observed high standard deviation. Light-cured composite resins enable better control of polymerization and lower technical requirements, which produces mechanical performance that remains consistent.

Clinical Implications for Pediatric Dentistry: The mechanical results from this study provide evidence-based material selection criteria for pediatric restorative dentistry. The high flexural strength of composite resins makes them suitable for posterior restorations that need to withstand chewing forces [29,30,31,32,33,34]. The restoration failure rate in primary-molar extensive Class I and Class II restorations becomes essential because restoration fractures represent the main reason for clinical failure [34,35]. The selection of materials for pediatric dentistry requires evaluation of various elements that extend past the basic requirement of mechanical strength. The flexural strength of conventional glass ionomer cements remains lower than that of other materials but they provide three essential benefits, which include tooth structure bonding through chemical adhesion, continuous fluoride emission, and safe interaction with dental pulp tissue [36,37,38]. The biological characteristics of glass ionomer cements make them suitable for particular dental uses. The dentist should perform the following: small-to-moderate Class I restorations in low-stress areas, Class V restorations in non-stress-bearing cervical regions of the mouth, interim therapeutic restorations in patients at a high risk of caries, and atraumatic restorative treatment (ART) in contexts with limited access to equipment; young patients who cannot cooperate with dental restorations would benefit from basic restoration methods. The intermediate mechanical properties of resin-modified glass ionomer cements make them suitable for applications that require both mechanical strength and biological benefits at moderate stress levels [39,40]. Recent systematic reviews and clinical studies published within the past five years have further highlighted the evolution of pediatric restorative materials. These include reports by Rashid et al. (2024) and Kalaskar et al. (2024), confirming that high-viscosity GICs and RMGICs remain clinically acceptable alternatives when biological advantages outweigh purely mechanical considerations. Similarly, Alqahtani et al. (2025) emphasized the shift toward bioactive and minimally invasive techniques that preserve tooth structure and enhance fluoride-mediated remineralization. The present findings align with these contemporary perspectives, supporting evidence-based material selection tailored to individual patient needs.

This study included seven specimens per group to detect major differences between the materials. Future research should increase the number of participants to detect subtle differences between materials. The complete assessment of restorative materials requires the testing of their mechanical properties, physical characteristics, and biological responses through wear resistance. The clinical value of these results will become established through additional in vivo studies and randomized clinical trials. The specimen dimensions used improved specimen handling and reduced the occurrence of edge fractures.

Limitations of this study include the in vitro design and the relatively small sample size per group, which may constrain the detection of small differences between the materials. Furthermore, flexural strength was the only parameter measured by the study, and non-mechanical and biological parameters were not measured in this research. Further studies involving larger sample sizes, extra mechanical properties, artificial aging protocols, and integrated mechanical–biological evaluation methods would provide a holistic perspective of restorative materials for pediatric dentistry on this issue.

## 5. Conclusions

The standardized comparative assessment revealed significant differences in flexural strength between different pediatric restorative materials that are commonly used. The findings suggest that composite resins work better for stress-bearing dental restorations in children’s teeth, but GICs and RMGICs perform best in situations with minimal-to-moderate stress because they maintain their best biological characteristics. The mechanical properties of composite resins make them suitable for posterior restorations in pediatric dentistry because they need to resist the forces of chewing. The clinical use of glass ionomer cement and resin-modified glass ionomer cement remains appropriate for particular situations because their biological properties including fluoride release, and chemical adhesion and biocompatibility, outweigh their lower flexural strength. These materials work well for three main uses, which include low-stress restorations, interim therapeutic interventions, and in uncooperative pediatric patients who need simplified techniques. The choice of materials for pediatric restorative dentistry should be guided by the biomechanical requirements of each clinical situation to achieve both structural strength and health benefits. Future research should focus on clinical validation tests and complete analysis of mechanical and biological properties, and long-term aging studies, to improve evidence-based material choices for pediatric dental care.

## Figures and Tables

**Table 1:** Restorative materials tested in the study.

Category	Material (Code)	Brand/Trade Name	Manufacturer	Type/Composition
Composite resin	FZ250	Filtek™ Z250	3M ESPE, St. Paul, MN, USA	Microhybrid composite
Composite resin	FS	Filtek™ Supreme XT	3M ESPE, USA	Nanocomposite
Composite resin	G	GC Gradia	GC Corporation, Tokyo, Japan	Microfilled hybrid composite
Composite resin	P	Premise	Kerr Corporation, Orange, CA, USA	Nanocomposite
Glass ionomer cement (GIC)	KM	Ketac™ Molar Easymix	3M ESPE, USA	High-viscosity GIC
Glass ionomer cement (GIC)	FIX	GC Fuji IX GP	GC Corporation, Japan	High-viscosity GIC
Resin-modified glass ionomer cement (RMGIC)	V	Vitremer	3M ESPE, USA	RMGIC with light-cure resin component

**Table 2:** Flexural strength values (MPa, mean ± SD) for all tested materials.

Category	Material (Code)	Flexural Strength (σ, MPa, Mean ± SD)	Range (MPa)
Composite resin	Filtek Z250 (FZ250)	32.1 ± 2.4 ^a^	27.3–36.9
Composite resin	Filtek™ Supreme XT (FS)	17.3 ± 1.8 ᵇ	15.1–20.2
Composite resin	Premise (P)	16.8 ± 2.0 ᵇ	14.2–19.5
Composite resin	GC Gradia (G)	8.2 ± 1.1 ᵈ	6.8–9.7
Resin-modified GIC	Vitremer (V)	12.5 ± 1.7 ᶜ	10.3–15.1
Glass ionomer cement	GC Fuji IX GP (FIX)	18.4 ± 3.2 ᵇ	14.5–23.1
Glass ionomer cement	Ketac™ Molar Easymix (KM)	4.3 ± 0.6 ᵉ	3.5–5.2

Note: Different superscript letters indicate statistically significant differences between materials (Tukey’s HSD test, *p* < 0.05). Normality and homogeneity tests: The Shapiro–Wilk test showed that all groups followed a normal distribution due to having *p*-values that exceeded 0.05.

## Data Availability

The original contributions presented in this study are included in the article material. Further inquiries can be directed to the corresponding authors.

## Abbreviations

The following abbreviations are used in this manuscript:

GICs	Glass ionomer cements
RMGICs	Resin-modified glass ionomer cements
FZ250	Filtek ^TM^ Z250
FS	Filtek ^TM^ Supreme XT
G	Gradia
P	Premise
KM	Ketac ^TM^ Molar Easymix
FIX	GC Fuji IX GP
V	Vitremer
